# Empathy, Theory of Mind, and Prosocial Behaviors in Autistic Children

**DOI:** 10.3389/fpsyt.2022.844578

**Published:** 2022-03-25

**Authors:** Xin Wang, Bonnie Auyeung, Ning Pan, Li-Zi Lin, Qian Chen, Jia-Jie Chen, Si-Yu Liu, Mei-Xia Dai, Jian-Hua Gong, Xiu-Hong Li, Jin Jing

**Affiliations:** ^1^Department of Maternal and Child Health, School of Public Health, Sun Yat-sen University, Guangzhou, China; ^2^Department of Psychology, School of Philosophy, Psychology and Language Sciences, University of Edinburgh, Edinburgh, United Kingdom; ^3^Autism Research Centre, Department of Psychiatry, University of Cambridge, Cambridge, United Kingdom; ^4^Guangdong Provincial Engineering Technology Research Center of Environmental Pollution and Health Risk Assessment, Department of Occupational and Environmental Health, School of Public Health, Sun Yat-sen University, Guangzhou, China; ^5^Department of Children's Healthcare and Mental Health Center, Shenzhen Children's Hospital, Shenzhen, China; ^6^Maternity and Children Health Care Hospital of Luohu District, Shenzhen, China

**Keywords:** autism spectrum disorder, prosocial behavior, empathy, Theory of Mind, Dictator Game

## Abstract

**Background:**

Previous research has suggested that children with autism spectrum disorder (ASD) display fewer prosocial behaviors, and the role of empathy or Theory of Mind (ToM) in prosocial behaviors of autistic children remains unclear.

**Methods:**

Data were obtained from an ongoing longitudinal study in Guangzhou, China. A total of 96 autistic children and 167 typically developing (TD) children were enrolled. Prosocial behaviors were assessed using a subscale of the Strength and Difficulties Questionnaire and Dictator Game (DG) paradigm with stickers as incentives. Empathic traits and ToM ability were measured using the children's Empathy Quotient and the Chinese version of ToM toolkit. Generalized linear models were used to assess the differences of prosocial behaviors and empathic traits, ToM ability between the two groups and the associations between empathic traits, ToM ability and prosocial behaviors in autistic children.

**Results:**

Compared with TD children, autistic children exhibited worse ToM ability and performed less pro-socially in the DG paradigm, while there were no differences regarding empathic traits. In autistic children, empathic traits especially affective empathy, were positively associated with parent-reported prosocial behaviors [β = 0.17, 95% confidence interval (CI): 0.07–0.27; β = 0.47, 95%CI: 0.33–0.60]. ToM ability was associated with DG paradigm (β = 1.03, 95%CI: 0.16–1.89).

**Conclusion:**

Autistic children showed less pro-sociality and ToM ability than TD children. In autistic children, empathic trait was associated with parent-reported prosocial behaviors while their ToM ability was associated with prosocial behaviors in experimental condition. Our findings indicated that better ToM ability and empathic trait might promote prosocial behaviors in autistic children.

## Introduction

Prosocial behaviors, which refer to actions that one engages in to benefit others, like helping, sharing, and comforting, is often believed to be the basis of human relationships (1). Children with autism spectrum disorder (ASD) might display fewer prosocial behaviors compared with typically developing (TD) children which are likely due to the social-communication deficits associated with an ASD diagnosis (2). For example, school-aged autistic children presented fewer prosocial behaviors in daily life than typically developing (TD) children when using parent- or teacher-reported rating scales (e.g., the Strength and Difficulties Questionnaire) (3, 4). However, in experimental settings, several studies have found that preschool autistic children do show helping behaviors (5, 6). In resource allocation tasks [e.g., Dictator Game (DG) and Ultimatum Game (UG)], autistic children showed a higher preference for equality than self-interest compared to TD children (7–10); but they tended to accept unfair offers (7, 10). Different measures might identify different situation of prosocial behaviors, resource allocation tasks such as DG is believed to be powerful to illuminate individuals' social interactions because it could examine the extent to which individuals attain their own goals while simultaneously displaying altruistic behavior toward unrelated individuals. However, directly measure of prosocial behavior in specific experimental environment may be lack of ecological validity (11). Therefore, it is particularly important to evaluate prosocial behaviors from different dimensions. In China, the study of prosocial behaviors in autistic children is limited and more studies are needed.

Empathy and Theory of Mind (ToM) are generally considered to be the major determinants of prosocial behaviors (12, 13). Empathy has been described as the ability to infer and share the emotional experiences with another (14), and consists of two components (15): the ability to understand the emotional state of others and distinguish another's feelings from one's own (i.e., cognitive empathy), and the ability to vicariously experience of the emotional experiences of others and respond emotionally in an appropriate way (i.e., affective empathy) (15). ToM, on the other hand, has been referred to as the attribution of mental states, such as emotion, desires, intentions and beliefs to others (16, 17). Given the theoretical similarities, cognitive empathy refers to a complex cognitive capacity, largely overlapping with ToM ability (12, 15). However, increasing studies have argued that ToM ability differs from empathy because ToM does not denote a sharing of another person's affective states, but rather a cognitive understanding of another person's mental states (18) (Supplementary Table S1 in Appendix A). ToM deficits have been considered as one of the major features in autistic children (19, 20). Studies have also shown impaired cognitive empathy but intact affective empathy in autistic children (21–24). Specifically, previous studies also indicated that there might be different roles of empathy or ToM ability on prosocial behaviors in children. Numerous researches have shown that prosocial behaviors, such as helping, sharing and comforting, are associated with preschool- and school-aged children's disposition to empathize with others (25–30). A meta-analysis of 6,432 children (2–12 years) revealed that children with advanced ToM abilities were more likely to show prosocial behaviors (*r* = 0.19), especially cooperating behaviors (*r* = 0.24) (13). When studying prosocial behaviors in relation to empathy and ToM simultaneously, Abrams et al. (31) found that empathy was positively associated with prosocial behaviors, but the association between ToM and prosocial behaviors was only shown in the non-competitive situation. Longobardi et al. (32) found that both empathic concern and ToM had direct positive effects on prosocial behaviors in primary school children. However, few studies focus on the associations between empathy, ToM and prosocial behaviors in autistic children with inconsistent results. For instance, a study of 20 school-aged autistic children and 20 language-age matched counterparts found that affective empathy was strongly related to peer interaction and prosocial behaviors (helping and sharing) in school-aged autistic children (33), while other studies found no associations between empathic responses and prosocial behaviors in preschool-aged and school-aged autistic children (34, 35). These earlier studies of autistic children with relatively small sample sizes only consider the role of empathy and ToM ability in prosocial behaviors separately, and to our knowledge, no studies have considered the role of empathy and ToM ability in prosocial behaviors simultaneously or defined them distinctly. In this study, we measured empathy and ToM simultaneously and evaluated prosocial behaviors via both parent-reported rating scales and experiments in autistic children and TD children in mainland China. We aimed to investigate the role of empathy or ToM ability in prosocial behaviors between these two groups.

## Methods

### Participants

We used the baseline data obtained from an ongoing study “the Guangzhou Longitudinal Study of Autistic Children” examining the developmental trajectories of 6- to 12-year-olds autistic children in Guangzhou, China. The participants were recruited between April, 2017 and February, 2020 from the Research Center of Children and Adolescent Psychological and Behavioral Development. Participants were included if they had a historical diagnosis of ASD which was confirmed by a combination of the Childhood Autism Rating Scale (CARS) and an expert clinician. Two professional child psychiatrists (Jin Jing and Xiu-Hong Li) further confirmed their diagnosis using Diagnostic and Statistical Manual of Mental Disorders, Fifth Revision (DSM-5) criteria. We also recruited a group of TD children at the same time, and the additional inclusion criteria for both groups were as follows: (1) chronological age between 6 years 0 months and 12 years 0 months; (2) voluntarily participation of the children's parents; (3) absence of dyslexia, attention deficit and hyperactivity disorder, emotional disorder and other disorders those would interfere with social ability; and absence of seizures, head trauma, cerebral palsy, or other movement disorders that would interfere with study assessments; and (4) absence of known genetic or chromosomal abnormalities or severe visual or hearing impairment. Only one child per family was recruited to ensure the independence of observations. If two or more children from one family were in the eligible age, we included the firstborn child (36). A total of *n* = 209 autistic children and *n* = 170 TD children were included in this longitudinal cohort. In this study, we selected a subsample of *n* = 96 autistic children (83 boys and 13 girls) and *n* = 167 TD children (97 boys and 70 girls) with complete questionnaire data, who were able to understand the instructions and able to complete all the behavioral assessments (detail of the inclusion flowchart was shown in Supplementary Figure S1 in Appendix A and comparison of demographic characteristics of included and excluded children was shown in Supplementary Table S2 in Appendix A).

## Measures

### Assessment of Prosocial Behaviors, Empathic Traits and Theory of Mind

Children's prosocial behaviors were evaluated via parent-reported rating scales and face-to-face experiments. The rating scale we used was the subscale of prosocial behavior in the Chinese version of the Strengths and Difficulties Questionnaire (SDQ). The subscale has five items rated on a 3-point Likert scale (0 = not true, 1 = somewhat true, 2 = certainly true). The total score of the five items ranges from 0 to 10 (higher scores represent more prosocial behaviors). The Chinese version of SDQ has been validated with a Cronbach's α of 0.73 for total score and 0.65 for subscale of prosocial behaviors (37).

Children's prosocial behaviors were also assessed using the Dictator Game (DG) paradigm (38), which has been validated in TD children aged 3–11 years in our previous study (39). In the DG paradigm, the dictator is given a windfall resource to allocate between him/herself and the virtual recipient who has no right to reject the offer. We arranged three settings to exclude factors that may affect children's decisions: (1) to avoid the influence of social distance, we set up a virtual anonymous character with the same gender and age as the participant (40); (2) to eliminate repeated interactions that might affect participant's willingness to share (i.e., in the repeated interaction settings, individuls may change his/her willingness to share by looking forward to the next round of feedback from the partner), we set up a one-shot interaction (40, 41); (3) to decrease the reputation effect caused by bystanders, we left the child alone in the room when he/she made decisions (42). We used stickers as allocated resources and prosocial behavior was measured based on the number of stickers that children shared and the decision to share or not. We also asked the children about their preference regarding the stickers by scores from 0 to 10.

The children's Empathy Questionnaire (EQ-C) was used to assess empathic traits. It has 27 items (13 reverse-scored items) rated on a 3-point scale: 2 = strongly agree, 1 = agree, 0 = disagree/strongly disagree (43). The Taiwan version was revised to 20 items (8 reverse-scored items) with three subscales of affective empathy, cognitive empathy and disrupt behaviors (44). We calculated the total score for all items and subscale scores for affective and cognitive empathy, and higher scores denoted stronger traits. The EQ-C and subscales have been validated with a Cronbach's α of 0.84, 0.69, 0.79, and 0.74 (44) (The detail of Taiwan version of EQ-C is shown in Supplementary Table S3 in Appendix A).

We used a set of ToM tasks which were adapted for use in China and have previously shown a Cronbach's α of 0.75 (45). Three subtasks were included: emotion attribution task, unexpected content task (46), and Sally-Anne task (47). In each subtask, children were marked with a pass when they gave the correct answer. We defined passing the ToM tasks when the children gave correct answers for all the three subtasks.

The detailed materials of the DG paradigm and the ToM tasks are provided in Appendix B of the Supplementary Material.

### Assessment of Covariates

Demographic information about children's age, gender, and being intervened formerly and/or currently, maternal age and education level, and per capita monthly household income was obtained via questionnaires.

Intelligence quotient (IQ) was assessed via the Chinese version of Wechsler Intelligence Scale for children, fourth edition (WISC-IV), which is suitable for children aged 6 years to 16 years 11 months. WISC-IV provides a full scale intelligence quotient (FSIQ) based on the sum of scores from the 10 core subtests, as well as four index scores: Verbal Comprehension Index, Perceptual Reasoning Index, Working Memory Index, and Processing Speed Index (48).

Severity of ASD symptoms was assessed via the Chinese Social Response Scale (SRS) which is a 65-item questionnaire used for children between 4 to 18 years. Each item is scored on a Likert scale ranging from 1 (not true) to 4 (almost always true). Total score was calculated ranging from 0 to 195, and higher scores indicated severe ASD symptoms. Cronbach's α coefficient for total scale wase 0.90 (49).

Considering that altruistic sharing behaviors might be somewhat constrained by the child's ability to inhibit control (50), we also assessed executive function by the Chinese version of Behavior Rating Scale of Executive Function (BRIEF) for children aged 6 to 18 years. The BRIEF is parent reported with 86 items using responses as follow: Never, Sometimes, or Often, coded as 1, 2, or 3, respectively. Higher score indicated greater perceived impairment of executive function, the subscales and total score were calculated and standardized into Z-score with the Cronbach α coefficient ranged from 0.74 to 0.96 (51).

### Procedure

Children underwent face-to-face measures performed by trained psychometrists, graduate students, or research assistants at the research center. Information on symptom severity, daily activities, executive functioning skills, and social cognitive abilities was obtained through in-person interviews with primary caregivers or validated tools/questionnaires. All the parents of the participants provided written consent. The study was approved by the Ethical Review Committee for Biomedical Research, Sun Yat-sen University (2015-No.29).

### Statistical Analyses

We calculated means and standard deviations for continuous variables and percentages for categorical variables. We compared the basic information between autistic children and TD children using chi-square tests and *t*-tests. We analyzed the correlations between all variables used in this study by using Pearson's correlation coefficient for two continuous variables, Cramer's V for two binary variables and Point biserial correlation coefficient for continuous variable and binary variable (see Supplementary Tables S4, S5 in Appendix A of supplement).

Generalized linear models were used to compare the differences of prosocial behaviors, empathic traits and ToM ability between the two groups, and to investigate the associations of empathic traits and ToM ability separately with prosocial behaviors in autistic children. We fitted crude models without any adjustments. We fitted adjusted models by adjusting for covariates including child's age, FSIQ, SRS total score, BRIEF total score, maternal age which were selected based on the correlation analyses. In order to eliminate the influence of sharing value due to the difference in sticker preference, we also adjusted for the degree of sticker preference when analyzing DG paradigm. Both empathic traits and ToM ability were entered into the same adjusted model to see whether they were independent of each other.

We conducted all statistical analyses with R 4.0.3 statistical software (R Core Team 2019). The results were presented as coefficient estimates (β) or odd ratios (OR) with a 95% confidence interval (CI). We considered a two-sided *P*-value < 0.05 as statistically significant.

## Results

The characteristics of the children were shown in Table 1. The mean age of the included autistic children was 7.4 ± 1.5 years and 86.5% of them were boy. Most of the autistic children were being intervened formerly and/or currently (72.9%). Compared with TD children, autistic children had lower FSIQ and scored higher on the SRS and BRIEF (*t* = −11.03, 18.99, and 9.50, all *P* < 0.01). Autistic children's mothers had low education levels with low household income compared with their counterparts (χ^2^ = 9.71 and 38.14, all *P* < 0.01). There were no differences between groups in child's age (*t* = 1.53, *P* = 0.11) or maternal age *t* = 0.57, *P* = 0.56).

**Table 1:** Demographic and Clinical characteristics of autistic children and TD children.

	**ASD (*N* = 96)**	**TD (*N* = 167)**	***P-*value^a^**
	***N* (%)/Mean (SD)**	***N* (%)/Mean (SD)**	
Age	7.4 (1.5)	7.1 (1.2)	0.11
Gender			**<0.01**
Boy	83 (86.5)	97 (58.1)	
Girl	13 (13.5)	70 (41.9)	
FSIQ	90.1 (18.3)	113.3 (12.6)	**<0.01**
CARS total score	30.9 (3.4)	-	-
SRS total score	89.4 (19.8)	43.7 (16.8)	**<0.01**
BRIEF total score	64.9 (8.7)	54.2 (9.0)	**<0.01**
Being intervened formerly and/or currently			-
Yes	70 (72.9)	-	
No	26 (27.1)	-	
Maternal age	29.9 (3.8)	29.7 (3.4)	0.56
Maternal education level			**<0.01**
Low (primary, secondary, high school)	44 (45.8)	45 (26.9)	
High (university and above)	52 (54.2)	122 (73.1)	
Per capita monthly household income			**<0.01**
Low (< ¥8,000)	57 (59.4)	36 (21.6)	
High (≥¥8,000)	39 (40.6)	131 (78.4)	

*ASD, Autism spectrum disorder; TD, Typically developing; SD, Standard deviation; FSIQ, Full-Scale Intelligence Quotient; CARS, Childhood autism rating scale; SRS, Social Response Scale; BRIEF, Behavior Rating Scale of Executive Function*.

a *The t-tests was used for the comparison of continuous variable while chi-square tests was used for the comparison of categorical variable*.

*The bold values meant the P -value was statistically significant*.

**Figure 1:**
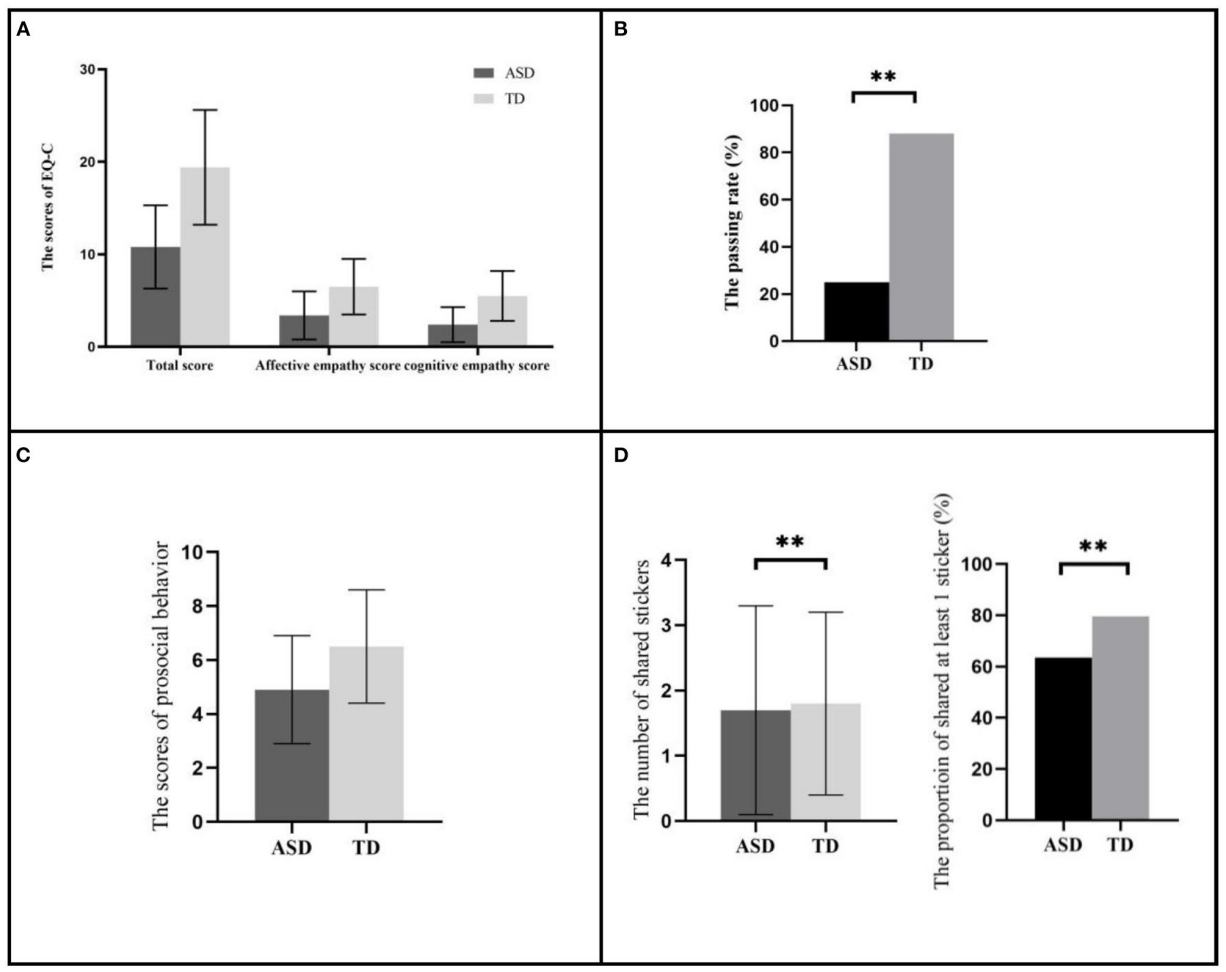
The comparison of empathic traits, ToM ability and prosocial behaviors between autistic children and TD children. **(A)** The comparison of EQ-C scores between the two groups. **(B)** The comparison of passing rate of the ToM toolkit between the two groups. **(C)** The comparison of prosocial behaviors score between the two groups. **(D)** The comparison of the number of shared stickers and the proportion of shared at least 1 sticker in DG paradigm between the two groups. All the comparison were adjusted for child's age, child's sex, FSIQ, SRS total score, BRIEF total score, maternal age, maternal education level and per capita monthly household income. The comparison of prosocial behavior in DG paradigm was further adjusted for degree of stickers preference. ASD, Autism spectrum disorder; TD, Typically developing; EQ-C, Children's version of Empathy quotient; ToM, Theory of mind. ^**^P < 0.01.

As shown in Figure 1 (detailed data shown in Supplementary Table S6 in Appendix A), after adjusting for potential covariates, there were no significant difference in EQ-C total score and the subscale score of affective empathy and cognitive empathy between autistic children and TD children (10.8 ± 4.5 vs. 19.4 ± 6.2, 3.4 ± 2.6 vs. 6.5 ± 3.0, 2.4 ± 1.9 vs. 5.5 ± 2.7; β = −1.96, 95%CI: −4.05~0.12, *P* = 0.06; β = −0.52, 95%CI: −0.27~-0.58, *P* = 0.40; β = −0.12, 95%CI: −1.06~0.82, *P* = 0.80). However, autistic children showed a lower passing rate in ToM toolkit compared to TD children (25.0% vs. 88.0%, *OR* = 0.10, 95%CI: 0.03–0.34, *P* < 0.01). There was no significant difference between groups in the score of prosocial behaviors in SDQ (4.9 ± 2.0 vs. 6.5 ± 2.1, β = 0.50, 95%CI: −0.36~1.36, *P* = 0.25). In DG paradigm, autistic children shared less stickers (1.7 ± 1.6 vs. 1.8 ± 1.4, β = −0.78, 95%CI: −1.42~-0.13, *P* = 0.02) with a lower proportion of sharing (63.5% vs. 79.6%, *OR* = 0.29, 95%CI: 0.09–0.88, *P* = 0.03) than those in TD children, and the frequencies were shown in Supplementary Table S7 in Appendix A.

Table 2 showed the associations between empathic traits, ToM ability and prosocial behaviors assessed by the SDQ subscale in autistic children. In the adjusted model, autistic children who had higher EQ-C total score and higher affective empathy score scored higher in the prosocial behavior subscale (β = 0.17, 95%CI: 0.07–0.27; β = 0.47, 95%CI: 0.33–0.60; all *P* < 0.001).

**Table 2:** Associations between empathic traits, ToM ability and parent-reported prosocial behaviors in autistic children.

	**The score of the prosocial behavior subscale (*****N*** **=** **96)**			
	**Crude model**		**Adjusted model^a^**	
	**Estimates (95%CI)**	***P-*value**	**Estimates (95%CI)**	***P-*value**
**EQ-C**
Total score	**0.21 (0.13, 0.29)**	**<0.001**	**0.17 (0.07, 0.27)**	**<0.001**
Affective empathy score	**0.52 (0.40, 0.64)**	**<0.001**	**0.47 (0.33, 0.60)**	**<0.001**
Cognitive empathy score	**0.27 (0.06, 0.48)**	**0.01**	0.06 (−0.18, 0.29)	0.65
**ToM**
Not pass	1 (Reference)		1 (Reference)	
Pass	0.50 (-0.44, 1.44)	0.30	0.27 (−0.76, 1.30)	0.61

*EQ-C, Children's version of Empathy quotient; ToM, Theory of mind; CI, confidence interval*.

a *Generalized linear models were used to investigate the associations of empathic traits and ToM ability with prosocial behaviors in autistic children. Crude model was fitted without any adjustment. Adjusted model was fitted with adjusting for child's age, FSIQ, SRS total score, BRIEF total score and maternal age*.

*The bold values meant the P -value was statistically significant*.

Table 3 showed the associations of empathic traits, ToM ability and prosocial behaviors assessed using the DG paradigm in autistic children. In the adjusted model, children who passed ToM toolkit shared more stickers compared to those who did not pass the tasks (β = 1.03, 95%CI: 0.16–1.89, *P* = 0.02). We did not observe significant associations of EQ-C total score and subscale score of affective or cognitive empathy with the number of shared stickers in autistic children.

**Table 3:** Associations between empathic traits, ToM ability and DG paradigm in autistic children.

	**The number of shared stickers (*****N*** **=** **96)**			
	**Crude model**		**Adjusted model^a^**	
	**Estimates (95%CI)**	***P-*value**	**Estimates (95%CI)**	***P-*value**
**EQ-C**
Total score	0.00 (−0.07, 0.07)	1.00	0.02 (−0.07, 0.11)	0.65
Affective empathy score	0.02 (−0.10, 0.15)	0.71	0.00 (−0.15, 0.15)	0.97
Cognitive empathy score	0.02 (−0.14, 0.20)	0.79	0.19 (−0.02, 0.39)	0.08
**ToM**
Not pass	1 (Reference)		1 (Reference)	
Pass	**0.97 (0.24, 1.71)**	**0.01**	**1.03 (0.16, 1.89)**	**0.02**

*EQ-C, Children's version of Empathy quotient; ToM, Theory of mind; CI, confidence interval*.

a *Generalized linear models were used to investigate the associations of empathic traits and ToM ability with prosocial behaviors in autistic children. Crude model was fitted without any adjustment. Adjusted model was fitted with adjusting for child's age, FSIQ, SRS total score, BRIEF total score, maternal age, and degree of sticker preference*.

*The bold values meant the P -value was statistically significant*.

The results were similar when both empathic traits and ToM ability were entered into the same adjusted model (Supplementary Table S8).

## Discussion

When using parent-reported measurements, we did not find differences in prosocial behaviors between autistic children and TD children; and empathic traits, especially affective empathy, was associated with prosocial behaviors in autistic children. However, we found poorer prosocial behaviors in autistic children than those in TD children when using experimental measurements, which were found to be associated with ToM ability.

Inconsistent results were previously obtained in three observational studies [one in Japan (3), two in the UK (4, 52)], indicating that parent-reported prosocial behaviors in 6–12 year-old autistic children were poorer than TD children. These earlier studies did not consider several important confounders (i.e., socio-economic and cognitive factors), while our study employed a large sample size with comprehensive information on these potential confounders. However, the inconsistency might have also resulted from cultural differences, such as culturally-specific norms and different socialization processes, thus affecting Chinese parents' evaluation of their children's prosocial behaviors (53). Unlike parent-reported measurements, we found that autistic children performed less pro-socially in experimental conditions. Similarly, several studies conducted in Western countries with small sample sizes found that autistic children can share things in resource allocation tasks (6, 10), but showed a higher preference for equality than self-interest compared to TD children (7, 54). In our study, prosocial behaviors measured by the DG paradigm is a form of strong reciprocity, which is a behavior that people may increase the fitness of unrelated individuals at a cost to themselves. This kind of prosocial behaviors is crucial for humans to establish cooperation (55, 56). In this paradigm, advanced social cognitive functions interact with others and an understanding of social norms are required when participants display prosocial behavior toward unrelated individuals (11, 55). Our results indicated that experimental measurements might be able to capture behavioral characteristics of prosocial behaviors in autistic children, and future studies should confirm our findings by using both parent-reported and experimental measurements.

When studying ToM ability and empathic traits simultaneously, we found that autistic children exhibited worse ToM ability but similar empathic traits than TD children. Consistent with previous studies, we confirmed that the ToM ability of autistic children was significantly lower than that of TD children (20, 57–59). However, autistic children had similar empathic trait compared to TD children, which was inconsistent with previous studies (43, 44, 60). When we further considered different types of empathy, we did not find differences in both affective and cognitive empathy. The results of affective empathy were consistent with most previous studies, indicating an intact affective empathy in school-aged autistic children (21, 23, 34, 61). However, parent-reported cognitive empathy in autistic children was not significantly lower than those in TD children, which was not in line with some of the previous findings (23, 61). This inconsistency might have resulted from the differences between self-reported questionnaires and behavioral assessments (21–23, 62). Moreover, most of the behavioral measurements of cognitive empathy were bundled together or conflated with ToM ability, resulting in the difficulties to distinguish cognitive empathy and ToM ability in these studies (21, 22). Fletcher-Watson and Bird (24) proposed that empathy can be broken down into four component stages: (1) noticing another's feeling; (2) correctly interpreting another's feelings; (3) feeling empathy; (4) responding in line with social norms. Although the subscale of cognitive empathy can reflect the second component of empathy, some of the items in this subscale might overlap with other components (i.e., item of 8 and 17 listed in the Supplementary Table S3 of Appendix A), limiting the power of this subscale. Therefore, more studies are needed to consider different components of empathy when studying the behavioral characteristics of autistic children.

In the DG paradigm, we found that ToM ability played an important role in the prosocial behaviors of autistic children. However, our previous study found that ToM ability did not contribute to prosocial behaviors of the DG paradigm in Chinese TD children, while other complex cognitive functions (i.e., inhibition control) may play a role in deciding resource allocation (39). In China, routinely enforced parental instruction that children should share things might eliminate the need for social insight to act as a trigger for sharing behavior for TD children in experimental conditions (13). Regrading autistic children, the development of social cognition is affected by the social motivation (63). Social Motivation Model suggested that early-onset impairments in social attention ultimately deprive the child of adequate social learning experiences, causing the imbalance in attending to social and non-social stimuli and subsequently disrupting social cognitive development (63). The resource allocation task in the context of game theory (i.e., DG paradigm) is developed based on the assumption that individuals can predict other people's actions when they understand others' motivations, preferences, and beliefs (18). Consequently, better ToM ability might contribute to prosocial behaviors of DG paradigm in autistic children. Our results might have clinical insights for behavioral interventions targeting social skills in real life since prosocial behaviors are of importance to social interaction, cooperation and adaptation (64).

In this study, we only observed a positive association between empathic traits and parent-reported prosocial behaviors in autistic children, and the associations were more pronounced when considering affective empathy. Autistic children might understand the emotions of others and respond appropriately since they have intact affective empathy (21, 34, 61). According to the Intense World Theory, autistic children might have hyper-perception and hyper-emotionality caused by hyper-functioning of local neural microcircuits (65). The vicariously emotional experience with others in autistic children might be intact or amplified, which could promote them to perform more prosocial behavior. Meanwhile, parents of ASD children tended to believe that their children behaved more pro-socially when they had higher affective empathic traits in Western countries (35). However, the results should be interpreted cautiously because empathy traits and prosocial behaviors in daily life were parent-reported, indicating the potential measurement error (34). Parents might report higher levels of empathic traits or prosocial behaviors due to exaggerated parental perceptions (66). One previous study in the Netherlands showed that the prosocial responses to peer distress were similar in autistic children and TD children in a computer task, indicating that the association between affective empathy and prosocial behaviors was less pronounced in experimental conditions (34), which was consistent with our findings. Since this is the first study to reveal the different role of empathic traits and ToM ability on prosocial behaviors in autistic children, more studies are needed to confirm our findings.

This study has several limitations. First, we used simple ToM toolkit instead of advanced ToM tasks, which might introduce a ceiling effect in TD children. However, our previous study has confirmed that most autistic children may have struggled with the advanced ToM tasks (20, 58, 59). Further research is needed to develop appropriate paradigms to compare ToM ability between autistic children and TD children. Second, we were not able to measure empathic traits and ToM ability by parent-report and experiment condition, which might introduce potential confounding of our findings. Third, we only performed one single task of the DG paradigm, and therefore it might be subject to measurement errors. Fourth, the behavioral characteristics of prosocial behaviors might be different when offering different resources (e.g., food, toys, attachment objects) (67). We only used stickers in this study as allocation resources although we have investigated the degree of sticker preference, and if the children disliked the stickers, we might not have been able to elicit prosocial behaviors. We found weak correlation between the prosocial behaviors measured by parent-report and by experiments (*r* = −0.10) which indicated that children might behave differently in their natural social environment than in experimental conditions. Future research is needed to study children's sharing behaviors of different resources in their natural environment. Fifth, we did not match on age and intelligence between the two groups despite of the use of statistical control in this study.

## Conclusions

Our study found that autistic children showed less pro-sociality and ToM ability than TD children. In autistic children, empathic trait was associated with parent-reported prosocial behaviors while their ToM ability was associated with prosocial behaviors in experimental condition. Our findings indicated that better ToM ability and empathic trait might promote prosocial behaviors in autistic children.

## Data Availability Statement

The raw data supporting the conclusions of this article will be made available by the authors, without undue reservation.

## Ethics Statement

The studies involving human participants were reviewed and approved by Ethical Review Committee for Biomedical Research, Sun Yat-sen University. Written informed consent to participate in this study was provided by the participants' legal guardian/next of kin.

## Author Contributions

JJ, X-HL, and J-HG: conceptualization, supervision, project administration, and funding acquisition. XW: methodology, formal analysis, and writing-original draft preparation. BA and L-ZL: study design and manuscript revise. XW, NP, QC, J-JC, S-YL, and M-XD: data collection. L-ZL: data curation. XW, BA, NP, QC, J-JC, S-YL, M-XD, J-HG, X-HL, and JJ: writing–review and editing. All authors contributed to the article and approved the submitted version.

## Funding

This work was supported by the Key-Area Research and Development Program of Guangdong Province (2019B030335001) and the National Natural Science Foundation of China (81872639, 82103794, and 82003482). BA was supported by the European Union's Horizon 2020 Research and Innovation Program under the Marie Skłodowska-Curie Grant Agreement No. 813546, the Baily Thomas Charitable Fund, and the Data Driven Innovation and the UK Economic and Social Research Council (ES/N018877/1) during the course of this work.

## Conflict of Interest

The authors declare that the research was conducted in the absence of any commercial or financial relationships that could be construed as a potential conflict of interest.

## Publisher's Note

All claims expressed in this article are solely those of the authors and do not necessarily represent those of their affiliated organizations, or those of the publisher, the editors and the reviewers. Any product that may be evaluated in this article, or claim that may be made by its manufacturer, is not guaranteed or endorsed by the publisher.
