# Supplementary material for: Empathy, Theory of Mind, and Prosocial Behaviors in Autistic Children
Source: Front Psychiatry. 2022 Mar 25;13:844578. doi: 10.3389/fpsyt.2022.844578 (PMC8990907; doi:10.3389/fpsyt.2022.844578)
Supplement: Supplementary file 1 [file Data_Sheet_1.pdf]

Supplementary Material

Appendix A

Table S1

Key Difference Between Empathy and ToM.

	Understanding another's mental state	Vicarious experience of the emotional experiences of others	Emotionally engaged in another's perspective
**Empathy**			
Affective empathy	×	√	×
Cognitive empathy	√	×	√
**Theory of Mind**	√	×	×

Figure S1 Flowchart of the study participants

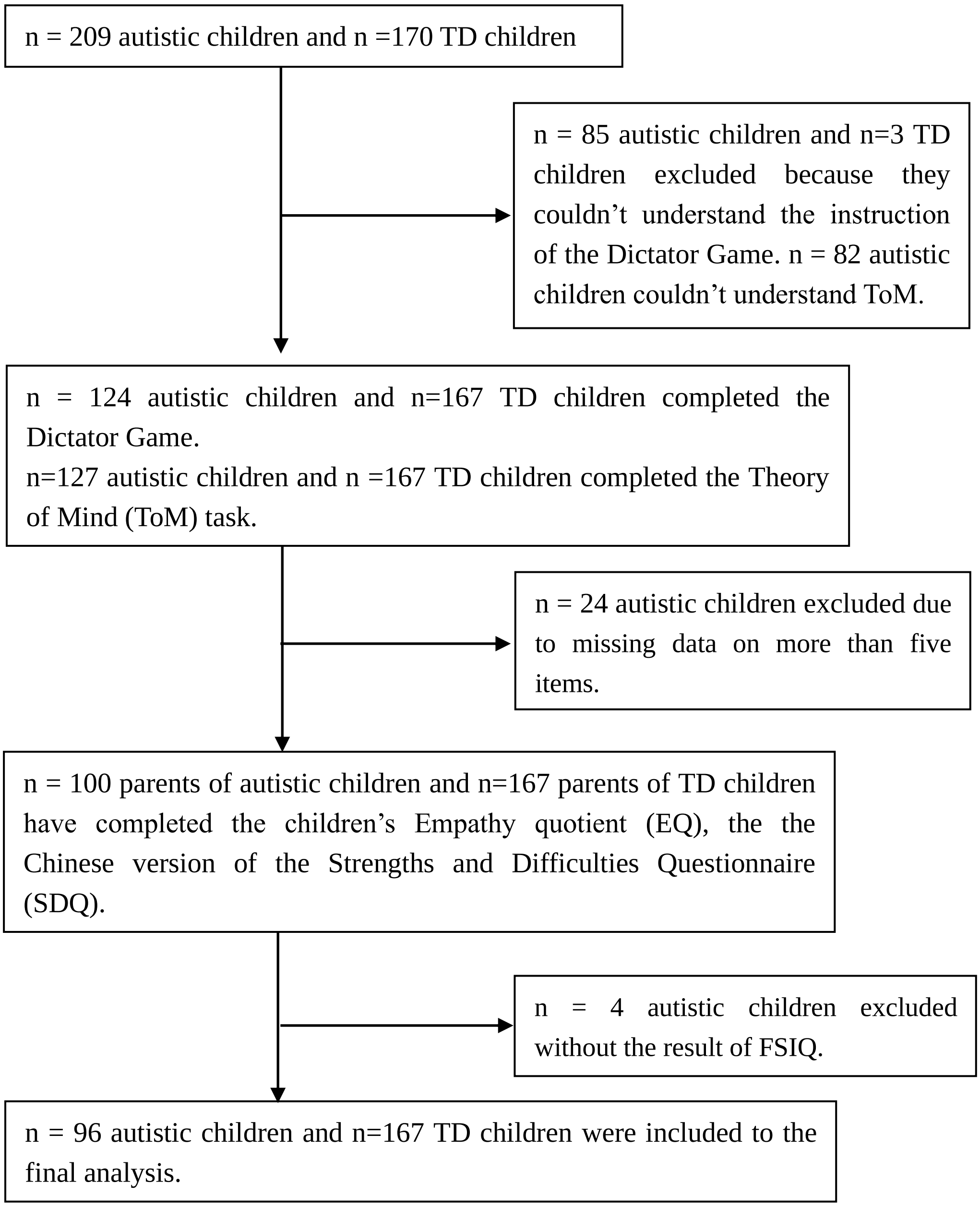

Table S2

Comparison of demographic characteristics of participants included in the final analysis and excluded from it

	ASD included (N=96) Mean (SD)/N (%)	ASD excluded (N=113) Mean (SD)/N (%)	*P* value	TD included (N=167) Mean (SD)/N (%)	TD excluded (N=3) Mean (SD)/N (%)	*P* value
Age	7.4 (1.5)	7.2 (1.4)	0.25	7.1 (1.2)	6.0 (0.0)	0.11
Sex			0.51			0.77
Boy	83 (86.5)	94 (83.2)		97 (58.1)	2 (66.7)	
Girl	13 (13.5)	19 (16.8)		70 (41.9)	1 (33.3)	
FSIQ	90.1(18.3)	82.3 (17.8) [a]	**0.02**	113.3 (12.6)	112.0 (4.2)	0.88
SRS Total score	89.4 (19.8)	99.4 (27.2)	< **0.01**	43.7 (16.8)	35.3 (10.0)	0.39
BRIEF Total score	64.9 (8.7)	66.6 (10.7)	0.23	54.2 (9.0)	54.0 (20.1)	0.98
Maternal age	29.9 (3.8)	30.1 (4.4)	0.70	29.7 (3.4)	33.0 (6.6)	0.10
Maternal education level			0.07			0.13
Low (primary, secondary, high school)	44 (45.8)	66 (58.4)		45 (26.9)	2 (66.7)	
High (university and above)	52 (54.2)	47 (41.6)		122 (73.1)	1 (33.3)	
Per capita monthly household income			0.08			0.62
Low (< ¥8000)	57 (59.4)	78 (70.8)		36 (21.6)	1 (33.3)	
High (≥¥8000)	39 (40.6)	31 (29.2)		131 (78.4)	2 (66.7)	

[a]: N = 41 (72 participants without result of FSIQ)

Table S3

The items of subscales of affective empathy and cognitive empathy(1, 2)

Subscale	Item
Affective empathy	My child likes to look after other people.
	My child would enjoy looking after a pet.
	My child shows concern when others are upset.
	My child gets very upset if they see an animal in pain
	My child would worry about how another child would feel if they weren't invited to a party
	My child gets upset at seeing others crying or in pain.
	My child likes to help new children integrate in class.
Cognitive empathy	My child often doesn't understand why some things upset other people so much.
	My child is quick to notice when people are joking.
	When playing with other children, my child spontaneously takes turns and shares toys.
	My child can be blunt giving their opinions, even when these may upset someone.
	My child listens to others' opinions, even when different from their own.
	My child can seem so preoccupied with their own thoughts that they don't notice others getting bored.
	My child can easily tell when another person wants to enter into conversation with them.
Disruptive behavior	My child has stolen something they wanted from their sibling or friend.
	My child is often rude or impolite without realizing it.
	My child blames other children for things that they themselves have done.
	My child sometimes pushes or pinches someone if they are annoying them.
	My child has been in trouble for name-calling or teasing.
	My child tends to resort to physical aggression to get what they want.

Table S4

The correlation matrix for empathic traits, ToM ability, potential covariates and prosocial behaviors in SDQ in autistic children [a]

	Prosocial behavior in SDQ	EQ total score	Affective empathy score	Cognitive empathy score	ToM
Prosocial behavior in SDQ	1.00				
EQ total score	0.46***	1.00			
Affective empathy score	0.66***	0.67***	1.00		
Cognitive empathy score	0.25*	0.58***	0.28**	1.00	
ToM	0.11	-0.19	0.01	-0.02	1.00
Child's age	0.08	-0.06	0.04	0.07	0.28**
Child's sex	0.11	0.08	0.01	0.03	-0.02
FSIQ	0.04	-0.17	0.00	-0.09	0.38***
SRS total score	-0.41***	-0.47***	-0.37***	-0.51***	0.01
BRIEF total score	-0.13	-0.29**	-0.21*	-0.23*	0.11
Being intervened formerly and/or currently	-0.01	0.01	0.05	0.12	-0.03
Maternal age	0.12	0.19	0.26*	-0.06	0.24*
Maternal education level	0.12	0.12	0.13	-0.09	-0.05
Per capita monthly household income	0.00	-0.09	-0.05	-0.17	0.06

[a]: Pearson's correlation coefficient examines the correlation of two continuous variable, Cramer's V examines the correlation of two binary variables, and Point biserial correlation coefficient examines the correlation of continuous variable and binary variable.

*<0.05, **<0.01, ***<0.001

Table S5

The correlation matrix for empathic traits, ToM ability, potential covariates and prosocial behaviors in DG paradigm in autistic children [a]

	Prosocial behavior in DG	EQ total score	Affective empathy score	Cognitive empathy score	ToM
Prosocial behavior in DG	1.00				
EQ total score	0.00	1.00			
Affective empathy score	0.04	0.67***	1.00		
Cognitive empathy score	0.03	0.58***	0.28**	1.00	
ToM	0.26*	-0.19	0.01	-0.02	1.00
Child's age	0.07	-0.06	0.04	0.07	0.28**
Child's sex	-0.09	0.08	0.01	0.03	-0.02
FSIQ	0.09	-0.17	0.00	-0.09	0.38***
SRS total score	0.20	-0.47***	-0.37***	-0.51***	0.01
BRIEF total score	0.03	-0.29**	-0.21*	-0.23*	0.11
Being intervened formerly and/or currently	-0.02	0.01	0.05	0.12	-0.03
Maternal age	0.20*	0.19	0.26*	-0.06	0.24*
Maternal education level	-0.11	0.12	0.13	-0.09	-0.05
Per capita monthly household income	0.09	-0.09	-0.05	-0.17	0.06

a: Pearson’s correlation coefficient examines the correlation of two continuous variable, Cramer’s V examines the correlation of two binary variables, and Point biserial correlation coefficient examines the correlation of continuous variable and binary variable.

*<0.05, **<0.01, ***<0.001

Table S6

The comparison of empathic traits, ToM ability and prosocial behaviors between children with ASD and TD children

	ASD (N=96) Mean (SD)/N (%)	TD (N=167) Mean (SD)/N (%)	P_{adjust} value [a]
EQ-C			
Total scores	10.8 (4.5)	19.4 (6.2)	0.06
Affective empathy scores	3.4 (2.6)	6.5 (3.0)	0.40
Cognitive empathy scores	2.4 (1.9)	5.5 (2.7)	0.80
ToM			**< 0.01**
Not pass	72 (75.0)	20 (12.0)	
Pass	24 (25.0)	147 (88.0)	
Prosocial behavior scores of SDQ	4.9 (2.0)	6.5 (2.1)	0.25
The number of shared stickers	1.7 (1.6)	1.8 (1.4)	**0.02**[b]
Shared at least 1 sticker or not			
No	35 (36.5)	34 (20.4)	**0.03[b]**
Yes	61 (63.5)	133 (79.6)	

Abbreviation:ASD, Autism spectrum disorder; TD, Typically developing; EQ-C, Children's version of Empathy quotient; ToM, Theory of mind; SD, Standard deviation; CI, confidence interval; SDQ, Strengths and Difficulties Questionnaire; REF, reference.

[a] The adjust model adjusted for child's age, child's gender, FSIQ, SRS total T score, BRIEF total T score, maternal age, maternal education levels, and per capita monthly household income.

[b] The comparison of the number of shared sticker and shared at least 1 sticker or not between group was further adjusted for the preference of the stickers.

Table S7

The frequency of each number of stickers shared by autistic children and TD children

	ASD N=96/N (%)	TD N=167/N (%)
Number of shared stickers		
0	36 (36.5)	34 (20.4)
1	13 (13.5)	47 (28.1)
2	12 (12.5)	35 (21.0)
3	28 (29.2)	37 (22.2)
4	2 (2.1)	6 (3.6)
5	3 (3.1)	6 (3.6)
6	3 (3.1)	2 (1.2)

Abbreviation:ASD, Autism spectrum disorder; TD, Typically developing.

Table S8 The associations of prosocial behaviors with empathy traits and ToM abilities when both of them were entered in the same model [a]

	The score of the prosocial behavior subscale				The number of shared stickers			
	Crude model		Adjust model		Crude model		Adjust model	
	Estimates (95%CI)	P value	Estimates (95%CI)	P value	Estimates (95%CI)	P value	Estimates (95%CI)	P value
EQ-C total score	**0.23 (0.15, 0.31)**	**<0.01**	**0.19 (0.08, 0.29)**	**<0.01**	0.02 (-0.05, 0.09)	0.62	0.06 (-0.04, 0.16)	0.25
ToM								
Not pass	1[Reference]		1[Reference]		1[Reference]		1[Reference]	
Pass	**0.95 (0.11, 1.78)**	**0.03**	0.63 (-0.38, 1.65)	0.22	**1.01 (0.26, 1.76)**	**<0.01**	**1.04 (0.13, 1.95)**	**0.03**

Abbreviation: EQ-C: Children's version of Empathy quotient; ToM, Theory of mind; CI, confidence interval.

a: Generalized linear models were used to investigate the associations of empathic traits and ToM ability with prosocial behaviors in autistic children. Crude model was fitted without any adjustment. Adjusted model was fitted with adjusting for child's age, FSIQ, SRS total score, BRIEF total score and maternal age.

Appendix B

Experimental materials of the DG paradigm and the ToM tasks
(English translation of the original version written in Chinese)

All the tests were conducted in a quiet and comfortable room. Two experienced graduate students (XW and QC) served as interviewers and were trained according to testing manuals. The participants were interviewed individually. A warm-up phase was arranged before the formal tests, during which the interviewers introduced themselves and talked with the child until the child seemed relaxed and ready to begin.

1. Dictator game

Pure altruism can be simulated by the dictator game (DG) (3). In this paradigm, the dictator is given a windfall resource to allocate between himself or herself and another player (the recipient), who has no right to reject the offer. The child was asked to select a favorite sticker from five types of stickers. Then, 6 identical stickers of the chosen type were spread out on the table. The child was told to decide how to allocate the stickers between him/herself and the unknown recipient, who was described as a child of the same age and sex (4). The child was instructed to put the stickers that the child wanted to keep for him/herself into an opaque bag with the child's name on it, and the stickers for the recipient were put into the other unmarked bag. The children's decisions were recorded after they left with their bags. Two variables were used to represent altruism: pro-socially (the decision to share or not) and level of prosocial behaviors (the number of stickers that the children shared).

1.1 Instructions

These stickers are gifts for you. They all belong to you now. A boy/girl (the same sex as the participant) of your age will come tomorrow. He/she may be your familiar classmate or a stranger you have never met, but we don't know who he/she is now. I have no time to give gifts tomorrow. And now, you can decide to give him/her some stickers or not. Remember that you can keep all the stickers for yourself. Of course, you can share some as you like. Put the stickers you want to keep for yourself in the bag with your name on it and put stickers you want to give away into the unmarked bag. In a minute I will leave, and the bag will be given to that child by another interviewer tomorrow. So no one will know your decision. Now I want to make sure that you understand the game. Who do these stickers belong to? Can you keep all the stickers? Where do you put the stickers you want to keep for yourself? Where do you put the stickers you want to give away? (Correct the child's answer if necessary)

Ok, I will leave now and you can start. Please open the door once you finish.

Alright, are you ready? Here we go. (the interviewer leaves)

1.2 Note

Sticker donations were not distributed to children who came later. This was done to avoid being influenced by the donations of the last child. Opaque paper bags were used to ensure

the confidentiality of children's decisions and to avoid responses designed to please the interviewer. Stickers were used as non-monetary incentives because they are highly valued among children and are widely used in previous studies (Benenson, Pascoe, & Radmore, 2007; Yongxiang, Liqi, & Zhe, 2013).

2. **ToM toolkit**

The Chinese version of the ToM toolkit includes three subtasks (5), the emotion attribution task, the unexpected content task, and the Sally-Ann task(6).

2.1 The emotion attribution task

In this task, the child would see a picture with a boy who is crying, and was asked "why does the boy cry?"

The correct answer could be "He was scolded by his mother", "He lost his way" and any other reasonable answers.

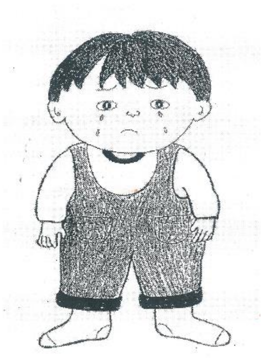

2.2 The unexpected content task

A pair of scissors were placed in a crayon box instead of crayons in the front of the child, and then the child was asked: "what will mom (a person who is not here) think is in the box?"

The correct answer is "crayons".

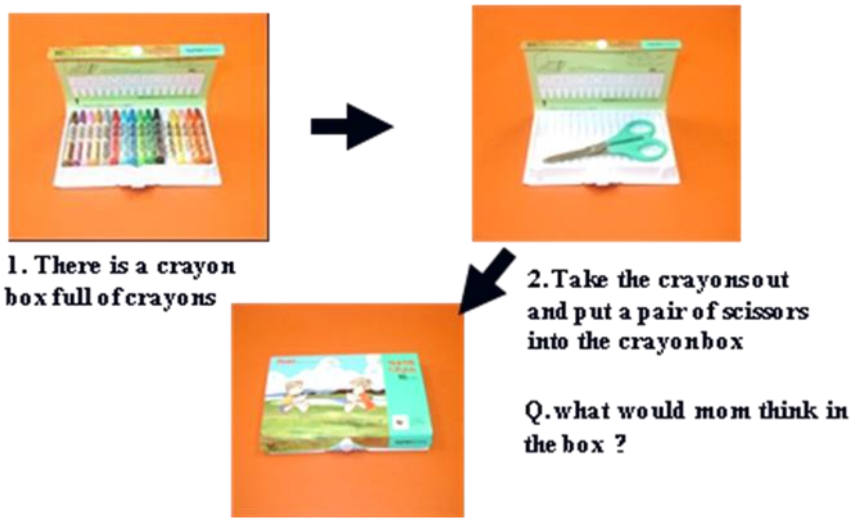

2.3 The Sally-Anne task

The interviewer manipulated props to simulate the scene while telling a story: A girl named Lily put her shoes into the cabinet in front of the house before she went in. Then a boy came and transferred the shoes to the box on the other side of the house. The core question was "Where will Lily go to find her shoes when she comes out?"

The correct answer is "the cabinet".

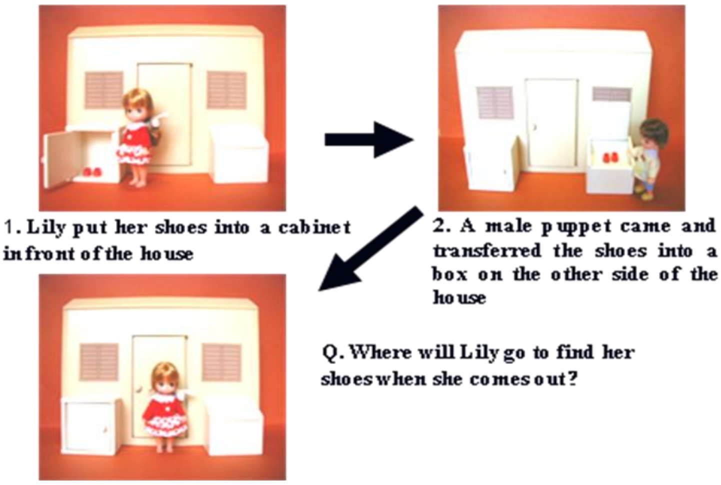

References

1. Huang H-Y. Psychometric Properties of the Children's Empathy Quotient and Systemizing Quotient (Unpublished master's thesis). Taiwan, China: National Taiwan University. 2015.
2. Auyeung B, Wheelwright S, Allison C, Atkinson M, Samarawickrema N, Baron-Cohen S. The children's Empathy Quotient and Systemizing Quotient: sex differences in typical development and in autism spectrum conditions. J Autism Dev Disord. 2009;39(11):1509-21.
3. Forsythe R, Horowitz, J. L., Savin, N. E., Sefton, M. Fairness in simple bargaining experiments. Games and Economic Behavior. 1994;6(3):347-69.
4. Blake PR, Rand DG. Currency value moderates equity preference among young children. Evolution and Human Behavior. 2010;31(3):210-8.
5. Qinying L. Study oftheory ofmind in Asperger's syndrome (Unpublished master's thesis). Guangzhou, China: Sun Yat-sen University. 2008.
6. Poletti M, Enrici I, Adenzato M. Cognitive and affective Theory of Mind in neurodegenerative diseases: neuropsychological, neuroanatomical and neurochemical levels. Neurosci Biobehav Rev. 2012;36(9):2147-64.
7. Perner J, & Wimmer, H. "John thinks that Mary thinks that…" attribution of second-order beliefs by 5- to 10-year-old children. Journal of Experimental Child Psychology. 1985;39:437-71.
8. Miller SA. Children's understanding of second-order mental states. Psychol Bull. 2009;135(5):749-73.
